# Safeguarding patients while implementing mechanical restraints: A qualitative study of nurses and ward staff’s perceptions and assessment

**DOI:** 10.1111/jocn.16249

**Published:** 2022-02-17

**Authors:** Liv Bachmann, Solfrid Vatne, Ingunn Pernille Mundal

**Affiliations:** ^1^ Department of Health and Social Sciences Molde University College Molde Norway; ^2^ Regional Centre for Child and Youth Mental Health and Child Welfare (RKBU) Department of Mental Health Faculty of Medicine and Health Sciences Norwegian University of Science and Technology (NTNU) Trondheim Norway

**Keywords:** assessment, nursing assessment, perception, psychiatric nursing, qualitative descriptive, restraint

## Abstract

**Aims and Objectives:**

To explore nurses’ and ward staff's perceptions and assessments of patient care while implementing mechanical restraints.

**Background:**

To prevent the risks associated with the use of restraints in psychiatry and ensure safe mental health care, it is necessary to know more about how the nursing staff experiences, comprehends and intervenes in managing patients subjected to coercive measures.

**Design:**

This study employed a qualitative descriptive design, in accordance with the COREQ guidelines.

**Methods:**

Semi‐structured interviews were conducted with 18 nurses and ward staff aged between 22 and 45 years old, who had experience implementing mechanical restraints. Data were digitally audio‐recorded and transcribed verbatim. Inductive thematic analysis was conducted using NVivo 12.

**Results:**

The participants believed that mechanical restraints should be used as a last resort and that safeguarding patients during implementation is important; however, their assessments of the patients’ physical and mental conditions varied. A clear difference emerged in how management qualified professionals handled situations prior to and during the implementation of mechanical coercive measures.

**Conclusions:**

The findings emphasise the need to focus on the assessment of patients prior to and during restraint, ensure the quality of safe implementation in a risk‐of‐harm situation, prioritise competence in education, and practice, and improve management.

**Relevance to clinical practice:**

The findings highlight the importance of assessing the physical and mental condition of patients while implementing restraints, as well as aiding the management, nurses and ward staff in tailoring safety procedures.

**What does this study contribute to a wider global clinical community?**

- The quality of safe implementation of restraints is lacking.
- Health authorities should establish national guidelines for the follow‐up of patients during mechanical coercive measures to ensure that nurses and ward staff are better prepared. There is a need for greater value and recognition of this aspect in practice.

## 1 INTRODUCTION

The use of coercion in mental health involves ethical, legal and clinical issues. Hence, reduction of restraint and safe mental health care are listed as objectives in the European Mental Health Action Plan (WHO, 2015). Recently, the use of restraints in mental health services has been included as one of the priority areas in the Norwegian patient safety program ‘In Safe Hands 24–7’ (Meld. St.^10^, ^2012‐2013^). Although the primary purpose of using restraints is to prevent unsafe behaviour, violence or self‐harm (Psykisk helsevernloven, 1999), it could cause additional risks to patients’ safety during the intervention (Thibaut et al., 2019). The use of mechanical restraints, pharmacological restraints or a combination of these two may potentially have deleterious physical and psychological consequences for patients (Chieze et al., 2019; Kersting et al., 2019), putting nurses and ward staff in a paradoxical situation, due to the risk of harm from the patients themselves.

In the international context, Norway has a relatively high rate of involuntary admissions in inpatient psychiatric departments (Salize & Dressing, 2004; Wynn, 2018). The Norwegian Directorate of Health reported a 12.7% increase in the number of involuntary admissions in 2020 compared to 2017, with 8,682 admissions related to mental health concerns. In 2020, 2,337 patients endured one or more approvals of coercive measures—a small increase of 0.7% compared to the number of patients who endured the same in 2019 (Norwegian Directorate of Health, 2021). The authorities’ goal of reducing restraints has not yet been reached. In relation to safe mental health care, it is important to look further into the practice of using restraints in psychiatry and the prevention of related risks. More knowledge about how nurses and ward staff experience, comprehend and intervene while managing patients subjected to coercive measures is needed.

## 2 BACKGROUND

The concept of ‘restraint’ involves several techniques and degrees of coercion and implementation. Internationally, ‘restraint’ in psychiatry is also known as ‘physical restraint’, which could be either manual or mechanical; chemical restraints are also addressed (Negroni, 2017).

A review highlights that no jurisdiction combines the full suite of laws, policies and practices that together might further the goal of eliminating coercion (Gooding et al., 2020). A study by Steinert et al. (2010) reports the incidence of mechanical coercive measures in psychiatric hospitals of countries such as Austria, Finland, Germany, Japan, the Netherlands, Norway, Spain and Switzerland. Mechanical restraints are forbidden for ethical reasons in countries such as the UK and Iceland (Steinert et al., 2010). Even though many countries allow the use of coercive measures, many countries work towards specific health policies that promote alternatives to coercive practice (WHO, 2021). In this context, several studies point to international initiatives to reduce the use of restrictive practices, such as the Six Core Strategies program, the Safe Wards Model (Fernández‐Costa et al., 2020; Goulet et al., 2017; Hirsch & Steinert, 2019), de‐escalation techniques and risk assessment (Fernández‐Costa et al., 2020). A Danish review (Bak et al., 2012) mentions three interventions that most likely reduce the number of mechanical restraints: cognitive milieu therapy, combined interventions and patient‐centred care.

In Norway, coercive measures are not regarded as part of treatment or therapy but as an extraordinary intervention in mental health care, which can only be applied to prevent patients from injuring themselves, assaulting others, or damaging buildings and physical objects and can be used regardless of patients` legal status when they are admitted to a mental healthcare facility. However, less restrictive interventions must first be proven unsuccessful. Mechanical restraint, according to the Norwegian Mental Health Care Act (1999, §4.8.), refers to different kinds of belts (i.e. restraint in a bed or outside of a bed for arms and feet only). According to the Norwegian legislation (Psykiskhelsevernloven, 1999), healthcare staff should continuously inspect patients subjected to mechanical restraints. While the legislation does not specify the competence level of the person who continuously assists the patient being restrained, the nursing staff mainly comprises mental health nurses, enrolled nurses, social educators, social workers and healthcare assistants.

The use of mechanical or pharmacological restraints—or a combination of the two—may have deleterious physical and psychological effects and outcomes in patients. A review found that the restraint position (e.g. the prone position used in Norway) can impede life‐maintaining physiological functions (Barnett et al., 2016). Physical injuries can cause respiratory and circulatory problems as well as nerve damages, more specifically through impaired consciousness, acute pulmonary oedema, respiratory arrest, loss of consciousness, aspiration and the release of stress hormones that may affect heart rate, rhabdomyolysis and thrombosis (Berzlanovich et al., 2012; Jönsson et al., 2018; Mohr et al., 2003; Nelstrop et al., 2006). Psychologically, coercive measures can activate emotions and have a retraumatising and traumatising effect (Cusack et al., 2018; Kersting et al., 2019).

Mental health nursing has traditionally focused on ‘interpersonal relations in nursing’ (Crenshaw & Peplau, 1952). Crenshaw and Peplau redefined the role of mental health nurses based on a more client‐focused perspective on what occurs in the interplay between the patient and the nurse and the therapeutic use of the professional self (Crenshaw & Peplau, 1952). The use of the professional self and assessments of patients is illuminated in a Danish study by Nielsen et al. (2018), who reported on forensic mental health clinicians’ experiences and assessments of the patients’ readiness to be released from mechanical restraints. The results highlighted the clinicians’ assessment of the quality of the alliance based on two parameters: (a) the patients’ insight into or understanding of the present situation and (b) the patients’ ability to maintain a good, stable relationship and cooperate with clinicians. These evaluations are included in the bigger picture of the assessments of patients’ readiness to be released from mechanical restraints (Nielsen et al., 2018). A recent qualitative study reported the impacts on the professional relationships when patients stopped engaging positively with healthcare professionals after restrictive practice, exacerbating the professionals` feelings of guilt (Mooney & Kanyeredzi, 2021). Today, the field of mental health nursing suggests different levels of assessments related to basic physiological needs, symptoms experienced (Barker, 2004), and elements such as risk, physical and mental status, symptomatology and effects of medication (Gamble & Brennan, 2005). A focus group study (Muir‐Cochrane et al., 2018) reported on the changing role in acute settings, focusing more on risk assessment and medication while attempting to practice trauma‐informed care.

A Canadian review of mental health care and practice revealed the lack of a systematic approach to patient safety (Brickell & McLean, 2011). Marcus et al. (2018) emphasised the need for research that examines the physical, emotional and psychological outcomes associated with patient safety events, stating that patient safety should become a key part of inpatient psychiatry (Marcus et al., 2018). In the Norwegian patient security program ‘In Safe Hands 24–7’, the use of restraints in mental health services was one of the priority areas that aimed to reduce the use of restraints in psychiatry. Countering action in the reduction and safer use of coercion, a national comprehensive training program ‘MAP’—Model for Aggression Prevention (SIFER, 2019), was introduced in Norway, aiming to direct health professionals to prevent and deal with aggression and violence to ensure the correct use of coercion in acute mental health care. To prevent poor health, evidence‐based clinical guidelines have been developed with recommendations for care, which will help healthcare professionals reduce the risk of harm for patients (Andersen Austegard et al., 2017; National Collaborating Centre for Mental Health, 2015). The National Collaborating Centre for Mental Health (NICE) guidelines imply that ward staff should monitor vital signs during the implementation of coercive measures and continue monitoring patients’ physical and psychological health for as long as clinically necessary. A local Norwegian guideline concerning mechanical coercive measures and their use in mental health care recommends that the care professional in charge assess the frequency of patient inspection conducted by nurses or social educators if unskilled worker monitor patients during restraints. The guidelines also recommend observing and controlling vital signs as well as assessing patients’ levels of consciousness and mental state (Andersen Austegard et al., 2017).

After an inspection of Norwegian acute psychiatric institutions in 2018, Ombudsman (2019) reported that the long‐term use of mechanical coercive measures gave rise to serious concerns related to severe somatic and psychological adverse effects in patients. The average duration of decisions on whether to use mechanical coercive measures was 11 h and 55 min, with a median duration of 6 h and 14 min (Ombudsman, 2019). A review by Cusack et al. (2018) exploring available evidence about the physical and psychological impact of restraints concluded that further research is required regarding mental health settings. Moreover, the researchers assumed that mental health nurses were in a prime position to use their skills for addressing the identified issues to eradicate the use of restraints and effectively meet the needs of those experiencing mental illness (Cusack et al., 2018). A systematic review by Douglas et al. (2021), reporting on the qualitative evidence from the perspective of patients experiencing physical restraints, identifies a bio‐psychosocial impact, in addition to an impact on therapeutic relationships and patients’ needs. The studies included mostly focused on the psychological impact, which was negative and connected to trauma or re‐trauma. A few studies have reported on the biological impact of pain and injury expressed through fear and distress. Conversely, studies addressing patients’ needs during the use of restraints, which highlighted the need for attention to patients’ comfort and safety, are sparse due to the practice of the restraint position and of asking the patient to sit up in order to breathe more easily (Douglas et al., 2021).

To prevent the risks associated with the use of restraints in psychiatry and ensure safe mental health care, it is necessary to know more about how the nursing staff experiences, comprehends and intervenes in managing patients subjected to coercive measures. Our literature review conducted using Medline, Ovid Nursing, PsychInfo and CINAHL indicates a limited number of studies on nurses` and ward staff's follow‐up of patients during the implementation of restraints.

## 3 METHODS

### 3.1 Aim

This study aimed to explore nurses` and ward staff's perceptions and assessments of patient care during the implementation of mechanical restraints.

### 3.2 Design

This study explored the clinical practice of mechanical restraint among nurses and ward staff using a qualitative approach with a descriptive design inspired by Sandelowski (Sandelowski, 2000). A qualitative description is amenable to obtaining straight answers to questions of special relevance to practitioners and entails the presentation of findings in the practitioner's everyday language. A qualitative descriptive design aims to produce findings close to the data (Sandelowski, 2010) and facilitates an exploration of the participants’ experiences and views that are relevant to glean rich descriptions of the phenomenon being studied (Polit & Beck, 2020). This study complied with the Consolidated Criteria for Reporting Qualitative Studies (COREQ) checklist (Tong et al., 2007; File S1).

### 3.3 Sample/Participants

We recruited a purposeful sample (Patton, 2015) of nurses and ward staff working at two acute psychiatric wards in two Norwegian hospitals. The inclusion criterion was having experience in the follow‐up of restrained patients. The participants (Table 1) were recruited by two nurse managers, with the aim of including those with maximum variations in terms of profession, specialised education in mental health care, sex, age and experience in the follow‐up of patients during the implementation of mechanical restraints. We estimated a sample of 15–20 participants as sufficient to gain information power and guided by Malterud et al.’s (2016) items and dimensions. The final sample was determined during the data collection process, with 18 participants assessed as having sufficient information power to match the aim of the study and the sample being dense enough for specificity (Malterud et al., 2016).

**TABLE 1:** Demographic details of participants

ID	Pseudonym	Profession	Specialized in mental health	Experience in acute psychiatric unit
1	Ariel	Nurse	Yes	5‐10
2	Blake	Nurse	No	<2
3	Charlie	Nurse	No	5‐10
4	Drew	Nurse	Yes	>15
5	Elliott	Nurse	No	<5
6	Finley	Nurse	Yes	>10
7	Hunter	Nurse	Yes	5‐10
8	Jordan	Nurse	Yes	<5
9	Karter	Nurse	Yes	5‐10
10	Lennon	Social Educator	No	<5
11	Milan	Social Educator	Yes	>5
12	Noah	Social Educator	No	<2
13	Parker	Social Educator	Yes	5‐10
14	Robin	Nurse Assistant	No	<2
15	Reese	Nurse Assistant	No	<2
16	Taylor	Nurse Assistant	No	<2
17	Sutton	Nurse Assistant	No	<2
18	Skyler	Nurse Assistant	No	5‐10

### 3.4 Data collection

Data were collected through semi‐structured individual interviews conducted between August and October 2020 by the first author, LB. As an introduction, LB presented herself as a PhD student with previous clinical experience in psychiatry. Each interview started with the question: ‘What types of situations can lead to the implementation of mechanical coercive measures?’ Participants were also asked about their experiences with observations and assessments of the patient under mechanical coercion and how they took care of the patient in such a situation. The interviews were digitally recorded and transcribed verbatim. Due to the COVID‐19 situation, interviews were conducted using Zoom, so that the interviewer and the participants could see and hear each other. The interviews lasted between 28 and 50 minutes. The interview guide was developed by the research team, which included all females with previous clinical experience in psychiatry. The interview guide was piloted in collaboration with educators from a university college who had previous experience in the follow‐up of patients being restrained within the context of acute psychiatric care.

### 3.5 Ethical considerations

The Norwegian Data Protection Official for Research (NSD) approved this study (reference number 503297). All participants received written and oral information about the requirements for participation in the study and the depersonalisation of the data; in addition, they were informed that participation in the project was voluntary, and they provided written informed consent to participate. Consent could be withdrawn at any time without giving any reason and without any negative consequences, and this would not influence the participants’ relationships with the staff. The interview guide was designed so that the participants could comment on the various topics without jeopardising their confidentiality.

### 3.6 Data analysis

Anonymised and verbatim transcribed interview data were uploaded to NVivo 12. An inductive, data‐driven thematic analysis process was chosen, based on four analytical phases, which were routed into a thematic analysis using six steps (Terry et al., 2017).

In Phase 1, ‘familiarizing yourself with your data’ became important due to the data collection process, with several interviews collected one after another, daily, using Zoom. Two professional librarians transcribed the audio files (*N* = 18) verbatim between October 2020 and January 2021. Meanwhile, the first author (LB) began listening to the audio files one by one. The purpose of doing this was to be acquainted with and gain insight into the dataset and to engage actively in the ‘observations’ by listening to the audio tapes, noticing questions coming up and writing memos while listening. At the end of the familiarisation phase, all transcripts were read, re‐read and discussed among the research team members (LB, SV and IPM). In this phase, the nurses and ward staff's descriptions of a highly complex and challenging situation and the unanimous view that implementing mechanical restraints ‘is the last resort we make’ became visible.

In Phase 2, we focused on ‘generating initial codes’ systematically across the entire data set, collating data relevant to each code. This phase was completed twice, ending with 176 semantic codes, implying that they captured the surface meaning of the entire dataset. Examples of coding were ‘justification and intention of coercion can be challenging in a difficult situation’, ‘deescalate situations and give the patient repeated opportunities to end behavior’, ‘have no system for one's own observations because every situation is so different’ and ‘the assessment starts with great intensity when the decision has been made to use compulsory medication and coercive measures’.

Phase 3 (following steps 3, 4 and 5 in Terry et al., 2017) involved collating the different semantic codes relevant to the potential semantic themes. In this process, we collated all the relevant coded data extracts from broader thematic levels, searching for relationships between themes (e.g. main overarching themes and sub‐themes within them). We reviewed and checked the themes in relation to the coded extracts through the entire dataset, eventually generating a thematic map, meaning that the research team interpreted contextual and culturally related sub‐themes that were unrelated to the sub‐themes connected to the assessment process. At the end of Phase 3, we refined the specifics of each theme and the overall story depicted in our analysis. Subsequently, for each individual theme (step 5 in Terry et al., 2017), we not only selected vivid, compelling extract examples relating to the research question and literature, but also identified the ‘stories’ that each theme conveyed in relation to the research questions.

### 3.7 Rigour

Lincoln and Gubas’s (1985) criteria for trustworthiness were used to establish rigour. The first author, LB, led the analysis and engaged in peer‐debriefing with co‐authors SV and IPM from the ‘familiarizing phase’ and throughout the analytic process to establish credibility (Lincoln & Guba, 1985). The researcher provided transferability through detailed descriptions of findings and verbatim quotes, aiming for readers from other care settings (Lincoln & Guba, 1985) to evaluate the extent to which the findings apply to new situations (Polit & Beck, 2010). Maintaining a research log of the study processes established dependability and confirmability (Lincoln & Guba, 1985).

## 4 FINDINGS

**FIGURE 1:**
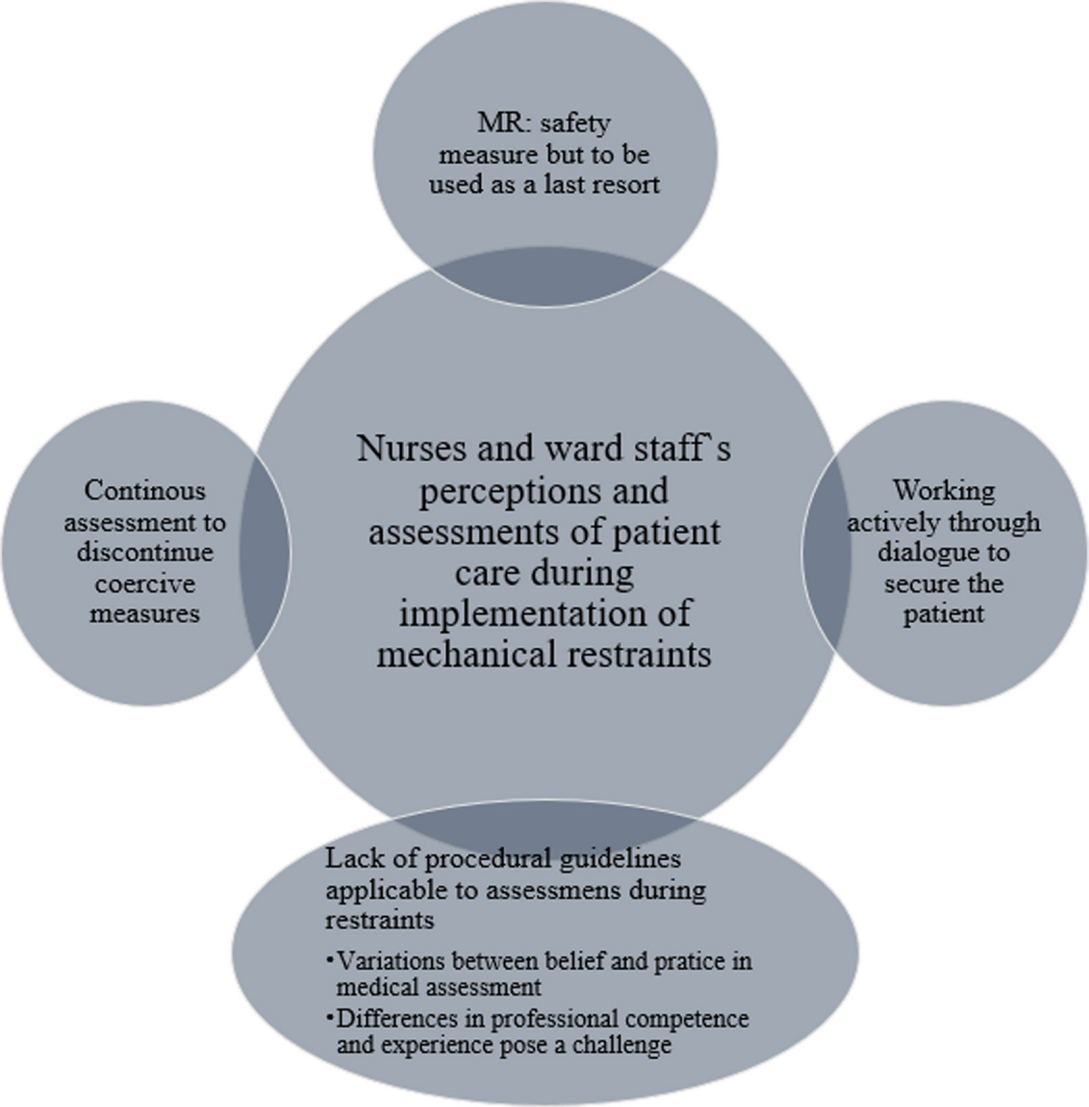
Illustration of themes

The study participants from two Norwegian acute psychiatric wards (*N* = 18) comprised nine nurses, four social educators and five ward assistants aged between 22 and 45 years old, whose experience of implementing mechanical restraints ranged from 1 to 16 years (Table 2). This analysis generated four main themes (Figure 1).

**TABLE 2:** Description of the actual sample

Characteristics	Participants	Nurses	Social educators	Nurse assistants	Mean (Range)
Age					34,5 (22–45)
Gender					
Male	9	3	1	5	
Female	9	6	3		
Education					
Bachelor	5	3	2		
<Bachelor	5				
Specialist Mental Health Care					
Yes	8	6	2		
Experience Acute psychiatric unit					5,7 (1–16)
Missing	2				
Duration interview					39 min (28–50)

### 4.1 Mechanical restraints: A safety measure but to be used as a last resort

Many participants, regardless of profession, stated that while mechanical restraints can be used as a safety measure in situations that escalate into violence, aggression or self‐harm, they should be used as a last resort. In most cases, there is a long duration between the starting point of agitation and the point where a patient must be placed under mechanical restraints. The participants expressed differing opinions regarding how different situations were handled, stating that each situation was assessed individually. One of the nurses explained mechanical coercion as being based on legal requirements for implementation.

It is very individual, but you must note the basis for the decision in terms of being dangerous to others, danger to oneself, or attack on others. Therefore, it is a kind of last resort; every option must be tried first. (Jordan, Nurse 8)

The participants also had a perception that mechanical coercion was not only negative, but also a possible measure that contributed to making the nursing staff experience security.

Some participants mentioned stricter norms for the implementation of coercive measures in the psychiatric treatment system; therefore, much effort was expended to avoid the use of mechanical coercion. The participants described it as a step‐by‐step approach, with a strong focus on identifying situations and the active use of de‐escalation and communication techniques in challenging situations. One of the participants conveyed how he resolved the escalating situation as follows:

First, we tried with a normal chat. Determine the underlying cause. Is there anything we can do to divert attention? Perhaps a distraction to the TV room helps or some form of regular medicine or a phone call to a relative. (Reese, Ward Assistant 2)

During the de‐escalation, the staff worked ‘closely’ with the patients over time. The use of verbal de‐escalation measures could also be based on what the patients themselves had stated in the interview upon admission to the ward. In such admission interviews, it is important to ask patients about what triggers them and how they want to be handled if they become particularly angry and agitated. Several of the participants also expressed the importance of providing patients with adequate space and time to be alone if de‐escalating techniques do not work. Another measure could imply replacing the staff who trigger the patients with others who have a better relationship with them. Actively involving the patient in de‐escalating options could also be a successful approach:

I remember a patient who acted on inventory (furniture and fixtures) and was threatening the staff. He was told to go to his room and calm down. Then, he intentionally aimed a blow at one of the staff, and we had to pin him to the floor and reveal what we experienced as threatening. So, we asked him if we could release him and together help him to calm down. ‘Do you think you can manage?’ And the patient did! (Charlie, Nurse 3)

When the staff experienced failure in the verbal process, the next step was to implement short‐term detention (manually holding the patient), isolation or the use of conveyor belts. Several participants described that the duration for the more lenient coercive measure—short‐term detention—had changed from a short timeframe of a few minutes to an entire hour. This led to discussions among the staff who experienced the situation and was perceived as a theme to be questioned. One participant raised the moral and legal aspects pertaining to the individual use of discretion of ‘short‐term detention’, how appropriate it was, and the need for decisions to end them. The reasons for ending short‐term detention depended on whether the patient calmed down, answered verbally, could communicate their wishes and promised to not harm the staff.

Moreover, the staff had reasons for not choosing a step‐by‐step approach and, instead, for quickly deciding to implement mechanical measures. They included the risk of serious self‐harm, earlier experiences with this patients` acting out behaviours, patients` intoxicated state during the admission situation, and explosive, potentially threatening situations. A participant stated:

Sometimes you know that the patient does not profit from short‐term detention. Maybe he has been abused or gone through many bad things—then we would rather go and get the mechanic restraint quickly. (Parker, Social Educator 4).

Apart from the descriptions of many elements that came into play in the assessment, the patients’ own wishes could affect the choice of coercive measure in terms of whether they prefer being ‘held’ for a long time or being directly restrained. The participants also stated that in their medical record, some patients explicitly conveyed their desire to use mechanical coercion instead of short‐term detention, which was cleared with ward management.

### 4.2 Working actively through dialogue to secure the patient

Once the decision on using mechanical coercion is made, and the patient is secured with a safety belt, the focus shifts from securing to further de‐escalation and gaining control of the situation. Many participants emphasised the importance of conveying information to the patient to ‘secure’ them and prevent further trauma. One of the participants described the importance of tuning in to the patients` words and employing suitable language:

It is in a way useful to try and reflect or describe how the patient appears, like, ‘Now you have become so angry, and we perceived you to be very threatening, and we do not want you to get into a situation where you are dangerous to yourself or others’. (Ariel, Nurse 1)

The same participant also described basic behaviours with which to secure the patient, such as meeting the gaze, actively breathing with the patient, being observant of one's own voice and position, and taking control of the situation alongside the patient.

Some of the participants indicated that the patient should not be exposed to a vulnerable situation and be the subject of others` observation while being restrained. To protect the patients from emotional harm, some offered a sheet to cover the patients when they were belted as a way of caring and showing dignity in a demanding situation. Another participant believed that patients then calmed down more quickly.

Several participants emphasised establishing a dialogue with the patient during belting. Some used the situation prior to the belting to start a conversation to which the patient could respond, which could then lead to an assessment of avoiding being belted for a longer period; this is important for reducing the sensation of being powerlessness when the patient is completely secured to a bed. The staff had to be receptive when listening to what the patient had to say, even though establishing a dialogue may be challenging. One participant stated:

You can comprehend that the patient is intoxicated or psychotic, and you are not able to establish a dialogue. It is challenging when you do not get through to the patient, and you do not get them to calm down. The patient is just as angry and aggressive, and all your efforts do not work. (Elliott, Nurse 5)

### 4.3 Lack of procedural guidelines applicable to assessments during restraint

At both institutions, the participants had undergone the MAP training program to understand, prevent, deal with, and follow‐up on aggressive and violent behaviours. This program aims to prevent violence and threats as well as the use of force and coercion. Many participants perceived that it was mandatory to participate in regular training to update their competence in line with current practices regarding how to handle such situations. The interviewer followed up on the participants’ descriptions and asked whether they had received guidelines or training beyond prevention, pertaining to how to check on patients during restraint. A major finding was the lack of awareness among several participants regarding guidelines available to nurses and ward staff about how to check on patients during mechanical coercion. They indicated that the department's procedures pertained to the doctors, who required statutory supervision of the patient immediately after coercive measures had been implemented. Among the participants, experiences of the frequency of ‘belt inspections’ varied from every half hour to every 7–8 h or were expressed as a continuous assessment through interactions between the doctor and the staff member observing the patient. One participant was more specific about belt inspections by physicians.

Doctors must have belt inspections when the patients have been placed on belts. First, it must be as soon as possible. Then, the doctors check everything: whether the belts are too tight, the circulation, respiration, confusion, or degree of disorientation of the patient, checking the skin, if there is edema, or the color and skin status. (Hunter, Nurse 7)

Many described the procedure for the nursing staff, which consisted of ‘continuous observation’, meaning that they had to continuously monitor the patient and carry a belt key if they needed to loosen the patient in unforeseen events. Participants also mentioned that staff should always hold the patient's hand. A nurse said that they were always two personnel to provide moral support to each other if the patient became angry and abusive.

Several participants expressed that the practice involved learning from each other, with continuous communication being a method of securing and making each other aware of what was particularly important in situations that required applying restraints. One participant described how he learned from other colleagues by observing how the nurses documented their progress in notes. This taught him what was mandatory to report after being bedside with patients under restraint. Another participant explained the procedure requirements for the nursing staff:

We have a quality system comprising procedures and guidelines for professional responsibility that can be used as a source for everything. However, it does not say exactly what I should do when I check up on patients during mechanical coercion. I do not think there are procedures for what to say and what to do. (Milan, Social Educator 2)

#### 4.3.1 Variations between belief and practice in medical assessment

Several participants described observations and assessments related to patients’ somatic health. One pointed to the medical gaze; reflections about the patient having gone through a hard fight prior to the implementation of the coercive measure and becoming drowsy after medication led the participant to describe himself as an intensive care nurse who had to be observant of the patient's respiration. The participant also described experiences with a previous patient who had lost consciousness after mechanical coercion, as a result of which the coercive measure had to be terminated quickly.

With regard to patients being under the influence of drugs, some participants indicated that it was important to monitor their awareness and determine whether they responded to pain stimuli. They also stated that it was critical to monitor respiration and linked this to whether the patient had received medication prior to, or during, mechanical coercion. Others linked respiratory problems to hyperventilation and patients expressing discomfort and claustrophobia; one of the participants mentioned that patients were often offered medication in such situations. Another reported being particularly concerned about the patients’ respiration, which could indicate an anxiety attack.

Some participants revealed that the first thing to keep in mind about patients’ basic needs is their circulation. Their observations were linked to whether the patients’ belts were too tight, which could affect the extremities, and some participants asked the patients whether their belts seemed tightened. Others expressed observations about skin colour and the importance of assessing patients’ temperature; this was linked to the situation prior to implementing the restraints as the patient could have been held in a ‘short‐term detention’ with 4–5 staff members trying to calm them down. Many perceived that the physician always came for statutory supervision, with the aim of double‐checking the circulation after the implementation of mechanical coercive measures.

Another observation expressed by several participants was that some patients wanted to go to the toilet immediately after being belted. They revealed the dilemma between releasing the patient in transport straps to go to the toilet and making the patient use a urine bottle or bedpan in the bed. Several of them also expressed that it was important to ensure that the patient received food and drinks. One said that he was afraid to give the patient food that could get stuck in the throat, and another mentioned that it was important to always observe the patient because they could have underlying diseases and a greater risk of developing a blood clot. One of the participants talked about how he constantly observed and assessed the patient's breathing, awareness and confusion but that the doctor was responsible for prescribing the frequency of measurements:

If we monitor vitals, the doctor decides how often we should take blood pressure, pulse, and respiration. Then, we, on our own initiative, check the patients` skin status related to circulation sometimes. (Hunter, Nurse 7)

Although several participants expressed how they followed up on the patients’ somatic condition during mechanical coercion, some particularly contrasting statements were made. When the interviewer summed up the description ‘about use of your clinical gaze’ and asked whether one of the participants had ever monitored blood pressure, counted pulse or respiration rate, the nurse responded that he never had been in a situation when he had felt it was necessary. Another stated:

If someone is restrained for a long time, then you have to take blood pressure and monitor them; but if we do not manage, then it is not that important; it is rare they are restrained for so long. (Drew, Nurse 4)

One of the ward staff explained his task as follows:

As soon as they are belted, the patient is checked carefully by a doctor for security reasons. So, my job is really to see if they are alive, just being there, and receive what’s coming verbally. It is part of their human rights to be allowed to speak out. (Taylor, Ward Assistant 3)

#### 4.3.2 Differences in professional competence and experience pose a challenge

Some participants stated that the formal responsibility of following up on the patients’ somatic condition lies with the nurse or social educator. They revealed that from a system perspective, both groups were equal and well trained to conduct patients’ somatic follow‐up. Professional competence and varying experience among the staff were two challenges of providing good intensive care in the follow‐up of patients when decisions on coercive medication and the use of coercive measures had been implemented. Several said that in combination with diagnosis and symptoms, holistic nursing competence (i.e. understanding the patients’ background and history and what happens to the body and brain) was a good professional ballast regarding not only detecting warning signals but also meeting the complexity of communication and handling challenging situations. Updated somatic competence was also expressed as being important. The participant with the shortest professional experience conveyed how he experienced contributing new skills to colleagues. By teaching others about systematic clinical examination and assessment of the patient, the participant realised that a colleague with more years of experience in psychiatry could gain new knowledge from him. Another commented on acute psychiatry as a system:

We have a lot to gain when it comes to competence development in psychiatry. It does not help with more heads or strong men. I do disagree that it is okay to use students instead of qualified nurses or social educators when patients are at their most vulnerable and in crisis. It must be recognized that we are engaged in intensive care. (Ariel, Nurse 1)

### 4.4 Continuous assessment to discontinue coercive measures

Several participants reported that their observations and assessments of the patient aimed for the coercive measure to have the shortest possible duration. Some described observations of patients’ behaviour, while others made assessments about the patient having ‘*calmed down*’ and not being angry anymore, with a lower severity of threats. Interactions with patients who were receptive to contact were also a part of their continuous assessment.

While some participants said that they tried to involve the patient by imparting information on how to proceed while being released from the restraints; others said that patients had to provide good contact, speak clearly and display calm body language before the belts could be loosened. Patients who did not provide appropriate emotional contact or voicing threats indicated that it was too early to end the restraint. Knowing the patient from previous admissions and being able to trust the patient had an impact on the assessment of whether participants could release them from the restraints. The lack of prior knowledge of the patient could mean less trust in the patient, which could lead to the patient remaining under restraints longer than necessary. Some participants said that they had previously misjudged whether the patient had calmed down enough, causing them to act out or commit self‐harm, which had led to the reintroduction of mechanical coercion. The discontinuing process, which follows a gradual method of releasing the patients by, for example, loosening the belt on an arm and a leg, takes place after close dialogue with colleagues who bear formal responsibility for the actual shift. Some participants described patients who calmed down with medication and eventually fell asleep, which was an indication that they could be released from the restraints. One of the participants stated, ‘*The challenge is that there will be a kind of subjective assessment of when to end the decision*. *There is no clear template or objective’ (Reese*, *Ward Assistant 2)*. Another described how the formal decision of determining of restraints was practised:

It is us, the ward staff, who decide when to release the coercive measure when we feel it is safe to do so. We then notify the doctor. Finally, we must write down how long the patient has been in belts. (Milan, Social Educator 2)

## 5 DISCUSSION AND IMPLICATIONS

This study sought to explore nurses` and ward staff's perceptions and assessments of patient care during the implementation of mechanical restraints. The findings conveyed their views on restraints as safety measures, but as a last resort. Through participation in the MAP training program, followed by frequent mandatory training, the participants revealed consistent views about their assessment process. The primary objective was to identify and deescalate situations through communication and avoid the use of coercive measures as far as possible by following a step‐by‐step approach. They worked actively to secure their patients through communication, fostering a caring relationship, and tried to ensure dignity during restraint implementation. They conveyed their opinion that only physicians controlled safe follow‐up procedures when patients were placed under restraints, and they were not aware of any professional guidelines applied to the nursing staff who continuously observed the patient under coercive measures. The findings also revealed that personnel training in observation and assessment during the implementation was not anchored at the system level but was left to experienced nurses or social educators who were responsible for providing in‐house training to their newly hired colleagues. We found variations among the participants in terms of their physical assessment and the extent to which they thought this was important. With regard to determining the coercive measures to be used, the assessment revealed individual discretion. Different professional competences and experiences related to the assessment process were highlighted as challenges.

### 5.1 Do we meet health authorities’ goals?

The objectives of the European Mental Health Action Plan include a reduction in the use of restraints and safe mental health care (WHO, 2015). The findings in this study revealed a well‐integrated view of coercive measures as a safety measure but as an absolute last resort in escalating situations. Thus, the care culture is aligned with international health policy goals that aim to reduce the use of coercive measures in accordance with legislation regulating acute mental health care in Norway (Meld. St.^10^, ^2012‐2013^; Psykisk helsevernloven, 1999; WHO, 2015) and in line with international initiatives to reduce the use of restrictive practices (Fernández‐Costa et al., 2020; Goulet et al., 2017; Hirsch & Steinert, 2019). Nevertheless, the findings may indicate that the management of acute mental health care has largely shed light on the quantified reduction in the use of coercive measures and, to a lesser extent, brought into focus the safe implementation of coercive measures.

#### 5.1.1 Intersection between goals, person‐centred care and legislation

The goal of reducing the use of coercion has been emphasised at the system level in acute mental health care, where MAP has been well established and comprehensively understood among the participants in this study. Within this picture, the person‐centred approach has particular significance through mapping, whereby patients note down what has triggered them in challenging situations and how they would prefer the care staff to act. Involving patients in their own situations is in line with a client‐focused perspective (Crenshaw & Peplau, 1952; SIFER, 2019). In other words, a client‐focused perspective, with repeated MAP training, can be understood as offering the professional caregiver another layer of relational and communicative competence. The introduction of the MAP program seems to have contributed to a more common attitude—an action clearly stated in the findings. As an integrated training program, MAP can help staff work systematically in their relational interactions with patients, contributing to robustness and security as a continuation of training, and focusing on ensuring effective communication.

Nevertheless, the findings show that the focus on reducing mechanical coercion had varying impacts on the participants. On the one hand, the participants were operating on the edge of the legislation, in that the duration of the less intrusive coercive measure of short‐term detention often lasted up to an hour. On the other hand, the participants could implement mechanical coercion faster if the patient requested this, based on previous experiences of abuse. This created a dilemma between the legislation (Psykisk helsevernloven, 1999) and the goal of reducing coercion (WHO, 2015). The participants also faced challenges balancing the need for ward safety while simultaneously providing person‐centred care (Muir‐Cochrane et al., 2018). Operating on the edge of legislation, the desire for trauma‐informed care and the likelihood of re‐traumatisation when deciding on the early use of mechanical coercion can be seen as a paradox and ethical challenge, balanced between promoting good and inflicting harm (Hem et al., 2018). The conflicting paradox when using coercion could lead to moral distress for healthcare professionals in their everyday practice (Hem et al., 2018).

#### 5.1.2 Imbalance in prepared competence before and during implementation

We obtained contrasting findings related to the preparedness of nursing staff prior to and during the implementation of mechanical restraints. MAP was rooted at the system level to reduce the use of coercive measures to prevent and deal with patients prior to a threatening situation. However, the findings indicate that professional measures for engaging in the safe monitoring of patients under mechanical coercive measures, such as guidelines, were absent at the system level, and assessments and decision‐making became an individual responsibility. Safe implementation is not only about prevention or a reduction in numbers and laws regulating the use of coercion. Content about how coercive measures should be performed should also play an important role, in relation to nurses and ward staff's professional responsibility in the assessment process. In 2020, 2337 patients in Norway endured one or more approvals of coercive measures (Norwegian Directorate for Health, 2021). Therefore, the quality of coercive measures should be assured at all levels and anchored in the management, from prevention in anticipation of a potential situation to the decision on implementation, the assessment process of the patient during the restraint, and the termination of the coercive measure is to be terminated, all of which should not be left to individual discretion.

Thus, it is relevant to ask whether only health service management is responsible for the safe follow‐up of patients under coercive measures, without considering the role that educational institutions play in preparing nurses and social educators to handle patient safety. In 2000, specialisation in mental health became an interprofessional educational course in Norway, based on a recovery‐oriented perspective and focusing on relational competence and user involvement. The transition from monodisciplinary to interprofessional education may be attributed, for example, to the fact that nurses have lost their deeper understanding of their subject‐specific competence, related to mental health nursing. Several of the participants emphasised the caring perspective, and we question whether the education policy may have failed to ensure more specific competence in high‐risk situations, as is required, for instance, when monitoring patients under mechanical coercion (Andersen Austegard et al., 2017; National Collaborating Centre for Mental Health, 2015).

It is paradoxical that although coercive measures are used to prevent harm to patients or ward staff, their use can inflict physical and mental harm to the patient. It is therefore important that nurses and ward staff exhibit broad competence in providing basic care to patients to prevent the risk of harm related to potentially deleterious physical and psychological effects and outcomes (Barnett et al., 2016; Berzlanovich et al., 2012; Cusack et al., 2018; Douglas et al., 2021; Jönsson et al., 2018; Kersting et al., 2019; Mohr et al., 2003; Nelstrop et al., 2006). The use of coercion in mental health involves ethical, legal and clinical issues. In our opinion, if nurses and nursing staff must implement coercive measures for safety purposes, the educational system and targeted mental health care must equip nursing staff with knowledge and skills through education, training observations and assessments of the implementation phase. If nurses and nursing staff gain increased competence, a focus on safe implementation may also contribute to reducing the use of coercion.

#### 5.1.3 Being unprepared led to scarce overall assessment

Our findings provide a basis for determining when mechanical coercive measures should be used. The participants revealed a focus on de‐escalation and on gaining control of the situation. Many also emphasised the importance of information to ‘secure’ the patient to prevent further trauma. These findings align with the traditional focus on mental health nursing and the therapeutic use of the professional self (Crenshaw & Peplau, 1952), which highlights the importance of paying additional attention to patients’ comfort during the implementation of restraints. Our findings reveal that the nurses were concerned about the patients’ well‐being during the implementation of restraints and that the participants had relational competences because of their repeated participation in MAP to update their knowledge and skills. Increased competence in dealing with the patients’ emotions may contribute to a less traumatic experience of being belted, which differs from the findings of a review by Douglas et al. (2021).

However, the findings revealed a lack of knowledge about guidelines and requirements for the follow‐up of patients and potential requirements for the participants’ own competence, in contrast to evidence‐based guidelines and recommendations (Andersen Austegard et al., 2017; National Collaborating Centre for Mental, 2015). A finding of concern is that nurses and ward staff considered patients’ safety to be solely the physicians’ responsibility. We question the professionals` own role in understanding and being aware of observations and early identification, which may be important for patients’ safety and general health in potentially dangerous situations (Barnett et al., 2016; Berzlanovich et al., 2012; Cusack et al., 2018; Douglas et al., 2021; Jönsson et al., 2018; Kersting et al., 2019; Mohr et al., 2003; Nelstrop et al., 2006). It may be relevant to identify who has the most competence in monitoring the patients’ physical and mental health condition in a potentially harmful situation. Updated somatic competence is critical, which is in line with our findings, as revealed by the nurse with the least experience teaching her more experienced colleagues about systematic clinical examinations and assessments. Acute mental health care should include this focus on communication and relational competence in demanding situations; by implementing a more comprehensive professional focus in the assessment of patients, nurses and others can also be able to take care of patients in accordance with the changed role (Muir‐Cochrane et al., 2018), requirements for assessments (Barker, 2004; Gamble & Brennan, 2005) and professional guidelines (Andersen Austegard et al., 2017; National Collaborating Centre for Mental Health, 2015). This can be important in determining when the coercive measures can be discontinued. The findings revealed that the criteria for whether and when the patient should be released were left to individual discretion, disclosed as a subjective assessment. This differs from the study by Nielsen et al. (2018), who reported on forensic mental health clinicians’ assessment regarding the patients’ readiness to be released from mechanical restraints, taking into consideration their insight into, or understanding of the present situation and their ability to maintain good and stable contact with and cooperate with clinicians.

We started the discussion with the question ‘Do we meet health authorities’ goals?’ Our study findings reveal problems pertaining to the need for broad competence and development in acute mental health care. The system level impact on individual professional caring for patients prior to and during the implementation of mechanical restraints is completely different. Cusack et al. (2018) call for research on the physical and psychological implications of physical restraints, assuming that mental health nurses are in a prime position to use their skills and knowledge in addressing the identified issues, with the aim to eradicate the use of restraints and better meet the needs of those experiencing mental illness. Muir‐Cochrane et al. (2018) report on their changing role as focusing more on risk assessment and medication, while attempting to practise trauma‐informed care. However, others have suggested assessments according to elements such as risk, physical and mental status, symptomatology, and effects of medication, in addition to the symptoms experienced (Barker, 2004; Gamble & Brennan, 2005).

How can we equip nurses and ward staff to meet contextual conditions? Leadership and management need to emphasise quality safety in all stages—not only in terms of prevention, but also when the decision is made in terms of assessment during the implementation and determination of the coercive measure. Our study findings indicate that acute mental health care has left the responsibility for the safe follow‐up of patients during mechanical coercion to individual nurses and ward staff. Regular training should be provided to improve competence and skills throughout the entire assessment process, prior to and during the implementation of restraints.

### 5.2 Limitations

This study has limitations. Considering the limited group of health professionals participating, their different professional roles and the fact that they were drawn from two limited geographical areas in Norway, the findings must be interpreted with caution. Despite these limitations, these nurses and ward staff contribute to the scarce empirical literature through insights into the practice of assessment and caring for patients under mechanical restraints—a subject area that is important for the development of health services in acute mental healthcare settings.

## 6 CONCLUSIONS

This study aimed to explore nurses` and ward staff's perceptions and assessments of patient care when implementing mechanical restraints. The findings emphasise the need for management—in addition to nurses and ward staff—to focus on the assessment of patients, both prior to and during the implementation of restraints. To ensure the quality of safe implementation in a potentially harmful situation, the focus should be placed on competence and professional responsibility. We suggest that nurses and ward staff should be well educated to contribute to the safer implementation of coercive measures, which also corresponds to an international call for integrated and coordinated holistic care, aiming to meet the patients` mental and physical healthcare needs. Nevertheless, this is related to a paradoxical situation that involves ethical, legal and clinical issues for patients and ward staff. Further research should explore how to develop nurses` and ward staff's assessments and follow‐up of patients under coercive measures, which is important for patient safety and clinical practice.

## 7 RELEVANCE TO CLINICAL PRACTICE

This study has several implications for clinical practice. First, the results highlight the paradox that although coercive measures are used to prevent harm to patients` and ward staff, its use may inflict physical and mental harm to patients`, but also lead to moral distress for healthcare professional in their everyday practice. Moreover, the results call attention to how the management qualifies professionals to handle situations prior to and during implementation of mechanical coercive measures. Finally, we stress the need for awareness of the patients` physical health in such situations.

## CONFLICTS OF INTEREST

None.

## AUTHOR CONTRIBUTIONS

LB, SV and IPM involved in study design. LB collected the data. LB, SV and IPM involved in analysis and manuscript preparation.

## Supporting information

Supplementary Material

## Data Availability

Research data are not shared.
